# *Leptospira* seroprevalence and associated risk factors in healthy Swedish dogs

**DOI:** 10.1186/s12917-022-03472-5

**Published:** 2022-10-22

**Authors:** Karolina Scahill, Ulrika Windahl, Sofia Boqvist, Lena Pelander

**Affiliations:** 1 Infection Medicine, 1 George Square, Biomedical Sciences, Edinburgh Medical School, College of Medicine and Veterinary Medicine, The University of Edinburgh, Edinburgh, EH8 9JZ UK; 2 Swedish National Veterinary Institute (SVA), 751 89 Uppsala, Sweden; 3 Department of Biomedical Sciences and Veterinary Public Health, Swedish University of Agricultural Sciences, Box 7039, 750 07 Uppsala, Sweden; 4 Department of Clinical Sciences, Swedish University of Agricultural Sciences, Ulls väg 26, 750 07 Uppsala, Sweden

**Keywords:** Canine, Seropositive, Saxkoebing, Microagglutination test

## Abstract

**Background:**

Leptospirosis is an emerging zoonotic infection worldwide and a cause of life-threatening disease in dogs. Seroprevalence in Swedish dogs is unknown. The aims of the present study were to estimate seroprevalence of pathogenic *Leptospira* in healthy dogs in Sweden using the microagglutination test (MAT) and a rapid point-of-care enzyme-linked immunosorbent assay (ELISA), and to evaluate risk factors of *Leptospira* exposure in Swedish dogs.

**Results:**

Positive MAT titres (≥ 1:50) were detected in 27/369 (7.3%) of included dogs. Five different serovars were represented of which the Saxkoebing serovar was the most common (64.3%), followed by Copenhagi (14.3%), Bratislava (10.7%), Icterohaemorrhagiae (7.1%), and Canicola (3.6%). The ELISA test (SNAP® Lepto) was positive in 3/316 (0.9%) dogs. Living in urban areas and contact with stagnant water were found to be risk factors for *Leptospira* seropositivity (*p* < 0.05) in a multivariable logistic regression model.

**Conclusion:**

In this first seroprevalence study of *Leptospira* in Swedish dogs, it was shown that healthy dogs without recent (24 months) travel history and antileptospira vaccination had been exposed to pathogenic *Leptospira interrogans* serovars. Contact with stagnant water and living in urban areas were independent risk factors for seropositivity.

**Supplementary Information:**

The online version contains supplementary material available at 10.1186/s12917-022-03472-5.

## Background

Leptospirosis is a zoonotic bacterial disease capable of transmission between a diverse group of animal species; while overrepresented in tropical regions, leptospirosis occurs worldwide [1, 2]. Dogs exposed to pathogenic *Leptospira* serovars risk developing life-threatening disease with a wide range of clinical signs [3, 4]. A recent meta-analysis concluded that adult male dogs with street access that come into contact with environmental water are at increased risk of *Leptospira* exposure [5]. As dogs often are exposed to environmental sources of infection through contact with soil and water, they may act as sentinels of pathogenic leptospires in the environment [5]. Several serovars are represented in dogs in Europe, and infecting serovars vary between different geographical regions [6, 7] which complicates vaccine distribution [8]. The inclusion of additional serogroups to antileptospiral vaccines for dogs have resulted in a marked decrease of disease incidence in a highly endemic area [9], and it is recommended that European dogs at risk should be vaccinated due to the possibility of zoonotic transmission and severe clinical course of disease [10].

Surveillance of canine leptospirosis is passive in Sweden. On average approximately twenty positive laboratory analyses are reported annually [11]. According to the Public Health Agency of Sweden less than one domestic human case per year is reported [12]. Most of the reported positive canine samples during the last ten years have been collected from dogs living in the counties of Stockholm, Västra Götaland and Skåne (Additional file 1). Serovars that have been detected in serum samples from Swedish dogs include *Leptospira interrogans* serovar (sv) Icterohaemmorhagiae*, Leptospira interrogans* sv. Canicola, *Leptospira interrogans* sv. Grippotyphosa, *Leptospira interrogans* sv. Bratislava, *Leptospira interrogans* sv. Saxkoebing, *Leptospira interrogans* sv. Sejroe and *Leptospira interrogans* sv. Grippotyphosa [11]. Rats in Sweden are confirmed to be reservoirs of *L.* Icterohaemmorhagiae, which is a serovar that commonly infects dogs in Europe [8, 13]. Moreover, the Swedish rat population is growing, which could contribute to an increased risk of environmental exposure of *Leptospira* [14]. The seroprevalence of *Leptospira* is not known in Swedish dogs, and surveillance has been encouraged to assess appropriate vaccination strategies [8]. Leptospirosis is not included in core vaccinations of dogs in Sweden.

The aims of the present study were to estimate seroprevalences of pathogenic *Leptospira* serovars in healthy dogs in Sweden using the microagglutination test (MAT) and a rapid point-of-care enzyme-linked immunosorbent assay (ELISA), and to evaluate risk factors of *Leptospira* exposure in Swedish dogs.

## Results

### Seroprevalence

Serum samples from 384 dogs were analysed with MAT. Fifteen samples were excluded due to contamination leading to inconclusive results, leaving a total of 369 samples (dogs) included in the study. Positive MAT titres (≥ 1:50) were detected in 27/369 (7.3%) samples (Table 1). One sample was positive to two different serovars (Table 2). In the county of Skåne 10/80 (12.5%) of samples were seropositive, in Stockholm 14/220 (6.4%), and in Västra Götaland 3/69 (4.3%) (Table 1). Five different serovars belonging to four serogroups were represented (Table 2), of which the Saxkoebing serovar was most prevalent (64.3%), followed by Copenhagi (14.3%), Bratislava (10.7%), Icterohaemorrhagiae (7.1%), and Canicola (3.6%). Copenhagi and Icterohaemorrhagiae serovars belong to the same serogroup (Table 4). The Saxkoebing serovar was the most prevalent serovar in all regions (Table 2), ranging from 40 to 100% of all positive samples. No other serovars than Saxkoebing were found in samples in Västra Götaland, and Skåne only had one other serovar (Bratislava), whereas the Stockholm region showed the highest variety of serovars (Table 2). Fourteen (50.0%) of seropositive samples had a titre ≥ 1:100, and titres ≥ 1:400 were only detected against the Saxkoebing serovar (Table 3). In Skåne 90% of titres were ≥ 1:100, and in Stockholm and Västra Götaland 23.8% and 33.3% had titres ≥ 1:100 respectively (Table 3).

**Table 1:** Titres and seroprevalence divided by regional area

**Titre**		**Stockholm (*n* = 220)**	**Västra Götaland** **(*n* = 69)**	**Skåne** **(*n* = 80)**	**All regions** **(*n* = 369)**
**1:50**	% (n)	39.3 (11)	7.1 (2)	3.6 (1)	50 (14)
	CI	23.6–57.6	2.0–22.7	6.3–17.7	32.6–67.4
**1:100**	% (n)	10.7 (3)	-	10.7 (3)	21.4 (6)
	CI	3.7–27.2		3.7–27.2	10.2–39.5
**1:200**	% (n)	-	3.6 (1)	14.3 (4)	17.9 (5)
	CI		6.3–17.7	5.7–31.5	7.9–35.6
**1:400**	% (n)	3.6 (1)	-	3.6 (1)	7.1 (2)
	CI	6.3–17.7		6.3–17.7	2.0–22.7
**1:1600**	% (n)	-	-	3.6 (1)	3.6 (1)
	CI			6.3–17.7	6.3–17.7
**Seropositive dogs**	% (n)	6.4 (14)	4.4 (3)	12.5 (10)	7.3 (27)
	CI	3.8–10.4	1.5–12.0	6.9–21.5	5.1–10.4

Number of tested dogs (n), relative prevalence (%), and 95% confidence interval (CI)

**Table 2:** Geographical distribution of positive serovars

**County**		**SAX**	**BRA**	**CAN**	**COP**^a^	**ICT**^a^
**Stockholm (*n* = 15)**	% (n)	40 (6)	13.3 (2)	6.7 (1)	26.7 (4)	13.3 (2)
	CI	2.0–6.5	3.7–37.9	1.2–29.8	10.9–52.0	3.7–37.9
**Västra** **Götaland (*n* = 3)**	% (n)	100 (3)	-	-	-	-
	CI	43.9–100				
**Skåne (*n* = 10)**	% (n)	90 (9)	10 (1)	-	-	-
	CI	59.6–98.2	1.8–40.4			
**All counties (*n* = 28**^b^**)**	n (%)	64.3 (18)	10.7 (3)	3.6 (1)	14.3 (4)	7.1 (2)
	CI	45.8–79.3	3.7–27.2	0.6–17.7	5.7–31.5	2.0–22.7

Number of individuals (n), relative prevalence (%), and 95% confidence interval (CI). Serovars: Saxkoebing (SAX), Bratislava (BRA), Canicola (CAN), Copenhagi (COP), and Icterohaemorhagiae (ICT)

^a^COP and ICT belong to the same serogroup: Icterohaemorhagiae

^b^One dog was positive for two serovars

**Table 3:** MAT titres of the different serovars

**MAT titre**		**SAX**	**COP**^a^	**BRA**	**ICT**^a^	**CAN**
**1:50**	% (n)	25 (7)	10.7 (3)	3.6 (1)	7.1 (2)	3.6 (1)
	CI	12.7–43.4	3.7–27.2	6.3–17.7	2.0–22.7	6.3–17.7
**1:100**	% (n)	14.3 (4)	3.6 (1)	3.6 (1)	-	-
	CI	5.7–31.5	6.3–17.7	6.3–17.7		
**1:200**	% (n)	14.3 (4)	-	3.6 (1)	-	-
	CI	5.7–31.5		6.3–17.7		
**1:400**	% (n)	7.1 (2)	-	-	-	-
	CI	2.0–22.7				
**1:1600**	% (n)	3.6 (1)	-	-	-	-
	CI	6.3–17.7				

Number of individuals (n), relative prevalence (%), and 95% confidence interval (CI). Serovars: Saxkoebing (SAX), Bratislava (BRA), Canicola (CAN), Copenhagi (COP), and Icterohaemorhagiae (ICT)

^a^COP and ICT belong to the same serogroup: Icterohaemorhagiae

### Rapid point-of-care test (ELISA)

The SNAP® Lepto test was used to analyse 316/369 (85.6%) of included samples (25/27 (92.6%) of MAT seropositive samples and 291/342 (85%) of MAT seronegative samples). Only 2/25 (8.0%) of the seropositive samples showed positive SNAP® Lepto results. The two SNAP® Lepto positive tests that were confirmed by MAT were both positive for serovar Saxkoebing with a titre of 1:50 and 1:200 respectively. One of the MAT seronegative samples showed a positive SNAP® Lepto result.

### Risk factor analysis

A total of 355/369 (96.2%) questionnaires were completed and were available for all but one (96.3%) of the 27 MAT seropositive dogs. Urban residency and contact with stagnant water (puddles, ditches) were significantly associated with *Leptospira* seropositivity (*p* < 0.05) in the univariable analysis and both factors were shown to be independent risk factors for *Leptospira* seropositivity in the multivariable analysis (Additional file 2).

## Discussion

In the present study 7.3% of healthy, non-leptospira-vaccinated dogs without a history of traveling outside of Sweden for the past 24 months had antileptospiral antibodies (≥ 1:50) to at least one serovar. Urban residency and contact with stagnant water were independent risk factors for *Leptospira* seropositivity. These results indicate that Swedish dogs, at least in the areas included in the study are exposed to pathogenic *Leptospira* serovars. The seroprevalence detected in the present study is lower in comparison to the global estimate of 18.5% [5], and in studies from continental Europe, where seroprevalences from 17 to 49% are presented [6, 7, 15, 16]. The seroprevalence (6%) of selected serogroups in Ireland [17] is similar to the Swedish average (7.3%), whereas regional seropositivity in Skåne (12.5%), in the south of Sweden, is similar to results presented from Greece (11.4%) and Thailand (12.1%) [18, 19]. Our results are not directly comparable to other studies due to a variation in titre cut-offs used to indicate seropositivity. Our chosen cut-off (≥ 1:50) is lower than the cut-offs used in some studies (≥ 1:100) [6, 20] and higher than that used in other studies (≥ 1:10) [7, 17]. These variations in choice of cut-off can result in both over-and underestimations of relative seroprevalence in the comparison between countries. Furthermore, in many studies health status and vaccination history of dogs are unknown, which potentially could contribute to an overestimated seroprevalence. A cut-off < 100 may increase the risk of false positives but is acceptable in exposure studies according to OIE guidelines [21].

The Saxkoebing serovar was predominant (64.3% of positive samples) in all included regions. The other detected serovars (Bratislava, Copenhagi, Canicola and Icterohaemorrhagiae) are all included in *Leptospira* vaccines registered for use in dogs but are likely to represent true bacterial exposure in this study, considering that none of the included dogs were vaccinated against leptospirosis. The Saxkoebing serovar has been detected in a wide range of wild animals such as foxes, brown bears and small mammals in Poland, Croatia and Austria [22–25]. It was also the second most prevalent serovar (24%) in dogs with clinical leptospirosis in a study from Southern Germany, and survival rate for Saxkoebing infection in that same study was 60% [26]. Moreover, Saxkoebing and the Sejroe serogroup is frequently encountered in the United Kingdom and in Germany [27, 28], but was the least common serovar found in a Spanish study [6]. Positive Saxkoebing antibodies have previously been found in Swedish dogs, but it is not known if they had clinical disease [11].

Our aim was to investigate if a point-of-care test could be used as a screening tool in seroprevalence studies. The SNAP® Lepto was chosen for this study because it detects antibodies against LipL32, a membrane protein of *Leptospira* that can be present in both acute and convalescent antibodies [29]. The SNAP® Lepto test was analysed in 92.6% MAT positive samples, but only 8% of these samples were positive on the SNAP® Lepto test. A previous study has shown a near 80% agreement of the SNAP® Lepto and MAT in dogs with titres ≥ 1:100 [30, 31]. Most of the dogs in this study had titres < 1:100 and did not have signs of clinical disease, which could explain the low agreement of the SNAP® Lepto and MAT. Furthermore, most dogs in this study were positive for serovars that were not included in previous studies evaluating the diagnostic performance of the SNAP® Lepto [30, 31]. The two positive SNAP® Lepto tests in this study were, however, MAT positive for serovar Saxkoebing and of low titres, 1:50 and 1:200 respectively, whereas one dog with a titre of 1:1600 (also Saxkoebing) was SNAP® Lepto negative. The single false positive SNAP result was not easily interpreted as the positive sample spot was weak compared to the control spot. It is possible that the correct interpretation of the SNAP test for this dog should have been negative. Similar difficulties in interpretation of the SNAP® Lepto has been reported in a previous study [32]. A single experienced laboratory technician performed the MAT, whereas the SNAP® Lepto tests were performed at the participating clinics by personnel of varying laboratory experience, which possibly could have contributed to the few positive SNAP® Lepto tests in this study. Point-of-care tests are, however, designed to be of easy use and most laboratory technicians are used to the technique, so this should not have affected the outcome significantly. The SNAP® Lepto test was designed to detect antibodies in clinical disease and not convalescent antibodies, which could explain the discrepancy with the MAT. Based on the results of this study the SNAP® Lepto test does not seem to be useful as a screening method in seroprevalence studies of healthy dogs.

Living in urban areas and contact with stagnant water were risk factors of *Leptospira* exposure in this study. This is consistent with results from a recent meta-analysis, which concluded that dogs with street access, and dogs that come into contact with environmental water had a higher risk of *Leptospira* exposure [5]. In that study, an increased risk of exposure was also seen in males, mixed-breed dogs and dogs over the age of four [5], but these variables were not associated with seropositivity in the dogs of the present study. However, risk factors for seropositivity varies between different studies [33–36], therefore leptospirosis should be considered a diagnosis in all dogs with compatible clinical signs [10].

Vaccination is recommended in areas with known risk of exposure [10, 37]. The present study has shown that dogs in Sweden are exposed to *Leptospira*, but available vaccines do not include protection against the predominant serovar Saxkoebing or other serovars within the Sejroe serogroup. The other serovars that were detected in the present study, Bratislava, Canicola, Copenhagi and Icterohaemmorhagiae, are, however, included in a quadrivalent vaccine that is registered for use in Europe.

### Limitations

Dogs were included in areas of Sweden where leptospirosis has been reported more frequently (Additional file 1). Hence, results might not be representative for the whole country.

## Conclusion

In this first seroprevalence study of *Leptospira* in Swedish dogs, it was shown that healthy dogs without recent (24 months) travel history and antileptospira vaccination are exposed to pathogenic *Leptospira interrogans* serovars. Contact with stagnant water and living in urban areas were shown to be independent risk factors for seropositivity. The results of this study could be used to promote awareness of leptospirosis among veterinarians and dog owners in Sweden and contribute to decisions of vaccine distribution in dogs.

## Methods

### Study population

A total of 384 healthy, privately owned dogs were included between October 2019 and June 2020. Informed consent was obtained from owners of all dogs. Most dogs were owned by staff working at the participating clinics, and some dogs were presented for routine visits and elective procedures. Dogs that had travelled outside of Sweden or had been vaccinated against leptospirosis during the past 24 months were excluded from the study, as were dogs showing signs of systemic illness. Sex, breed and age was known in 368/369 (99.7%) of included dogs. Ages ranged from 3 months to 16 years (median 4.0; interquartile range 2–7). Sex distribution was 119/368 (32%) female, 58/368 (16%) female neutered, 116/368 (32%) male, and 75/368 (20%) male neutered. The majority of dogs (84%) were purebred. In total 88 breeds were represented. The most common breeds were Labrador retriever (8%), Golden retriever (6%), Flat Coated retriever (5%), German shepherd (4%), and Staffordshire bull terrier (4%).

### Study area and study design

**Fig. 1:**
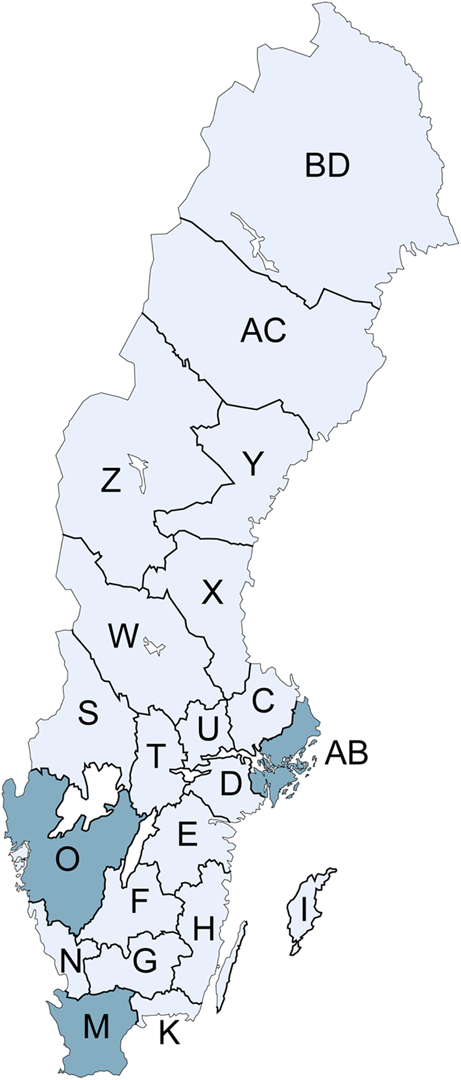
Map of Sweden. Shaded areas show sampling locations. AB: Stockholm, O: Västra Götaland, M: Skåne

This cross-sectional seroprevalence study was conducted at 17 veterinary clinics and hospitals in the Swedish counties of Stockholm, Västra Götaland and Skåne (Fig. 1). These counties were chosen due to a high incidence of previously notified canine leptospirosis cases (Additional file 1). Most samples (60.1%) were collected in Stockholm. The remaining samples were collected in Västra Götaland (18.5%) and Skåne (21.4%). Nine dogs from Uppsala, a county bordering to Stockholm, were also included in the study. The sample size (*n* = 384) for apparent seroprevalence was based on epidemiological calculations using Epitools (Ausvet, Australia) estimating a prevalence of 50% with a precision level of 5%.

### Data collection

#### Questionnaire

Dog owners were asked to complete a semi-structured questionnaire, written in Swedish, at the time of blood sampling (Additional file 3). The questionnaire contained a mix of open-ended questions and dichotomous questions stating age, sex, breed, residential environment, vaccination and travel history. Data collected included possible contact with wildlife, rats, and whether the dog was used for hunting. Owners were also asked about presence of other animals in the household and whether the dog had been in contact with stagnant water (puddles, ditches), sea water or lakes (Additional file 3).

#### Rapid point-of-care test (ELISA)

Blood samples were collected by venipuncture of the cephalic vein into sterile serum-separating tubes. Blood was left to clot for at least 30 min and then centrifuged.

A commercial rapid point of care test, the SNAP® Lepto test (IDEXX Laboratories Europe, Hoofddorp, The Netherlands) was analysed by veterinarians or laboratory technicians at the respective clinics on the day of sample collection according to the manufacturer’s instructions [38]. The SNAP® Lepto test detects both IgG and IgM antibodies to serovars Grippotyphosa, Canicola, Pomona and Icterohaemorrhagiae [30, 38]. Remaining serum was stored in cryogenic test tubes at -20 °C until they were transported to the NVI.

#### Microscopic agglutination test

Serum samples were transported in batch and stored at -20 °C at the National Veterinary Institute until MAT analysis, which was performed in accordance with standard protocols [21, 39] by an experienced laboratory technician. Samples were tested for presence of antibodies to a panel of ten serovars (Table 4). The strain Mus 2A is a domestic strain so far detected in Swedish mice, pigs and dogs [40, 41]. Titres < 1:100 might be considered as evidence of previous exposure according to OIE guidelines [39]. A titre of ≥ 1:50 was therefore defined as positive regarding previous exposure to *Leptospira* in the present study.

**Table 4:** Species, serovar, strain and serogroups used as antigens for the microscopic agglutination test (MAT)

Species	Serovar	Strain	Serogroup
*L. interrogans*	Icterohaemmorhagiae	Kantorowicz	Icterohaemmorhagiae
	Canicola	Hond Utrecht IV	Canicola
	Autumnalis	Akiyamai A	Autumnalis
	Pomona	Pomona	Pomona
	Bratislava	Jez	Australis
	Australis	Ballico	Australis
	Copenhagi	M20	Icterohaemorrhagiae
*L. kirschneri*	Grippotyphosa	Duyster	Grippotyphosa
*L. borgpetersenii*	Sejroe type istrica	Mus2A	Sejroe
	Saxkoebing	Mus 24	Sejroe

### Data analysis

Microsoft Excel 2016 and JMP Pro 14 (SAS Institute, Cary, North Carolina) were used for statistical analysis. Epitools (Ausvet, Australia) was used for sample size calculation and to determine confidence intervals for sample proportion. Fischer’s exact test was used for univariable analysis of independent risk factors and MAT seropositivity as an outcome. All variables with a *p*-value of < 0.2 were included in a multivariable logistic regression model with MAT seropositivity as the dependent variable, and a value of *p* < 0.05 was considered significant (Additional file 2).

## Supplementary Information

**Additional file 1: Appendix 1.** Shows number of reported dogs to the Swedish Board of Agriculture (2010-2020) [42]. All laboratory positive samples (antibodies (>1:100) and PCR) are reported regardless of clinical suspicion of disease. Travel and vaccination history is not known in tested dogs.

**Additional file 2: Appendix 2.** Shows risk factors, number and percentage of exposed individuals for both seronegative and seropositive dogs. A Fischer’s exact test with a confidence level of 95% has been used to calculate the p-value in the right columns. Logistic regression was applied in the multivariable model.

**Additional file 3: Appendix 3.** Shows the questionnaire that was answered by the dog owners (translated from Swedish).

## Data Availability

The datasets analyzed during the current study are available from the corresponding author on request.

## Abbreviations

ELISA: Enzyme-linked immunosorbent assay
CI: Confidence interval
IgG: Immunoglobin G
IgM: Immunoglobin M
MAT: Microagglutination test
NVI: National Veterinary Institute
OIE: World Organization for Animal Health
OR: Odds ratio
P: *P*-Value
SMHI: Swedish Meteorological and Hydrological Institute
