# Supplementary material for: Leptospira seroprevalence and associated risk factors in healthy Swedish dogs
Source: BMC Vet Res. 2022 Oct 22;18:376. doi: 10.1186/s12917-022-03472-5 (PMC9587587; doi:10.1186/s12917-022-03472-5)
Supplement: Supplementary file 1 — Additional file 1: Appendix 1. Shows number of reported dogs to the Swedish Board of Agriculture (2010-2020) [42]. All laboratory positive samples (antibodies (>1:100) and PCR) are reported regardless of clinical suspicion of disease. Travel and vaccination history is not known in tested dogs. [file 12917_2022_3472_MOESM1_ESM.docx]

| **County** | **2010** | **2011** | **2012** | **2013** | **2014** | **2015** | **2016** | **2017** | **2018** | **2019** | **2020** | **Total (%)** |
| --- | --- | --- | --- | --- | --- | --- | --- | --- | --- | --- | --- | --- |
| Stockholm | 2 | 2 | 10 | 5 | 2 | 6 | 3 | 8 | 2 | 4 | 5 | 49 (19.4) |
| Uppsala | 1 | 3 | 1 |  |  |  |  |  |  |  | 2 | 7 (2.8) |
| Södermanland |  | 1 |  |  |  |  | 1 | 2 |  |  |  | 4 (1.6) |
| Östergötland |  |  |  |  |  |  |  | 1 |  |  | 2 | 3 (1.2) |
| Jönköping | 2 |  | 2 |  |  | 2 | 1 |  |  | 1 | 2 | 10 (4.0) |
| Kronoberg |  |  |  |  | 1 |  |  |  |  |  | 2 | 3 (1.2) |
| Kalmar |  | 1 | 1 |  |  |  |  |  |  |  |  | 2 (0.8) |
| Gotland |  | 1 |  |  |  |  |  |  | 1 | 3 | 3 | 8 (3.2) |
| Blekinge | 1 |  | 1 |  |  |  |  |  |  |  |  | 2 (0.8) |
| Skåne | 6 | 4 | 6 | 8 | 2 | 5 | 7 | 12 | 5 | 12 | 13 | 80 (31.7) |
| Halland | 1 |  |  |  | 1 | 1 | 1 | 1 |  | 1 | 1 | 7 (2.8) |
| V.Götaland | 1 | 1 | 8 | 3 | 1 | 4 | 1 | 11 | 4 | 8 | 15 | 57 (22.6) |
| Värmland | 2 |  |  |  |  |  |  |  |  |  |  | 2 (0.8) |
| Örebro |  |  |  |  |  | 2 | 1 |  | 1 |  | 1 | 5 (2.0) |
| Västmanland |  | 1 |  |  |  | 1 |  | 1 |  | 1 |  | 4 (1.6) |
| Dalarna |  | 1 |  |  |  |  |  |  |  |  |  | 1 (0.4) |
| Gävleborg |  |  |  |  | 1 |  |  |  |  |  |  | 1(0.4) |
| Västernorrland | 1 |  | 1 |  |  |  |  |  |  |  |  | 2 (0.8) |
| Jämtland |  |  |  |  |  |  |  |  |  |  | 2 | 2 (0.8) |
| Västerbotten |  |  |  |  |  |  |  |  |  |  |  | n/a |
| Norrbotten | 1 | 1 |  |  |  |  |  |  |  |  |  | 2 (0.8) |
| Unknown |  |  |  |  | 1 |  |  |  |  |  |  | 1 (0.4) |
| **Total** | 18 | 16 | 30 | 16 | 9 | 21 | 15 | 36 | 13 | 30 | 48 | 252 |
