# Supplementary material for: Leptospira seroprevalence and associated risk factors in healthy Swedish dogs
Source: BMC Vet Res. 2022 Oct 22;18:376. doi: 10.1186/s12917-022-03472-5 (PMC9587587; doi:10.1186/s12917-022-03472-5)
Supplement: Supplementary file 2 — Additional file 2: Appendix 2. Shows risk factors, number and percentage of exposed individuals for both seronegative and seropositive dogs. A Fischer’s exact test with a confidence level of 95% has been used to calculate the p-value in the right columns. Logistic regression was applied in the multivariable model. [file 12917_2022_3472_MOESM2_ESM.docx]

| **Variable** | **Number of dogs** | **Seropostive % (n)** | **95% CI** | **Univariable analysis** | | | **Multivariable analysis** | | |
| --- | --- | --- | --- | --- | --- | --- | --- | --- | --- |
|  |  |  |  | **OR** | **CI** | ***p*** | **OR** | **CI** | ***p*** |
| **Age (n=368)** |  |  |  |  | | |  | | |
| >5 years | 170 | 8.8 (15) | 5.4-14.1 | 1.5 | 0.7-3.4 | 0.3 | - | | |
| <5 years | 198 | 6.1 (12) | 3.5-10.3 |  | | | - | | |
| **Sex (n=368)** |  |  |  |  | | |  | | |
| Female | 177 | 7.3 (13) | 4.3-12.2 | 1.0 | 0.5-2.2 | 1.0 | - | | |
| Male | 191 | 7.3 (14) | 4.4-11.9 |  | | | - | | |
| **Breed**  **(n=368)** |  |  |  |  | | |  | | |
| Pure | 311 | 7.7 (24) | 5.2-11.2 | 1.5 | 0.5-6.5 | 0.55 | - | | |
| Mixed | 57 | 5.3 (3) | 1.8-14.7 |  | | | - | | |
| **Residential area**  **(n=355)** |  |  |  |  | | |  | | |
| Urban | 214 | 9.8 (21) | 6.5-14.5 | 3.0 | 1.1-9.0 | **0.025** | 3.3 | 1.2-9.1 | 0.0218 |
| Rural | 141 | 3.6 (5) | 1.5-8.0 |  | | |  | | |
| **Drinking outside**  **(n=355)** |  |  |  |  | | |  | | |
| yes | 273 | 7.3 (20) | 4.8-11.0 | 1.0 | 0.4-2.8 | 1.0 | - | | |
| no | 82 | 7.3 (6) | 3.4-15.0 |  | | | - | | |
| **Contact with stagnant water (puddles, ditches)**  **(N=355)** |  |  |  |  | | |  | | |
| yes | 57 | 15.8 (9) | 8.5-27.4 | 3.1 | 1.3-7.3 | **0.016** | 3.5 | 1.5-8.5 | 0.0053 |
| no | 298 | 5.7 (17) | 3.6-8.9 |  | | |  | | |
| **Swimming in lakes**  **(n=355)** |  |  |  |  | | |  | | |
| yes | 217 | 8.3 (18) | 5.3-12.7 | 1.5 | 0.6-3.7 | 0.4 | - | | |
| no | 138 | 5.8 (8) | 3.0-11.0 |  | | | - | | |
| **Hunting**  **(n=355)** |  |  |  |  | | |  | | |
| yes | 24 | 12.5 (3) | 4.3-31.0 | 1.9 | 0.4-6.4 | 0.3 | - | | |
| no | 331 | 7.0 (23) | 4.7-10.2 |  | | | - | | |
| **Rats in area**  **(n=355)** |  |  |  |  | | |  | | |
| yes | 241 | 5.8 (14) | 3.5-9.5 | 0.5 | 0.2-1.2 | 0.1 |  |  |  |
| no | 114 | 10.5 (12) | 6.1-17.5 |  | | |  | | |
| **Contact wild animals**  **(n=355)** |  |  |  |  | | |  | | |
| yes | 175 | 6.9 (12) | 4.0-11.6 | 0.9 | 0.4-2.0 | 0.7 |  | | |
| no | 180 | 7.8 (14) | 4.7-12.6 |  | | |  | | |
| **Other dog in household**  **(n=355)** |  |  |  |  | | |  | | |
| yes | 163 | 8.6 (14) | 5.2-13.9 | 1.4 | 0.6-3.2 | 0.4 |  | | |
| no | 192 | 6.3 (12) | 3.6-10.6 |  | | |  | | |
