# Supplementary material for: Leptospira seroprevalence and associated risk factors in healthy Swedish dogs
Source: BMC Vet Res. 2022 Oct 22;18:376. doi: 10.1186/s12917-022-03472-5 (PMC9587587; doi:10.1186/s12917-022-03472-5)
Supplement: Supplementary file 3 — Additional file 3: Appendix 3. Shows the questionnaire that was answered by the dog owners (translated from Swedish). [file 12917_2022_3472_MOESM3_ESM.docx]

| **QUESTIONNAIRE** | | |
| --- | --- | --- |
| 1. Age |  | |
| 2. Sex (entire/castrated) |  | |
| 3. Breed |  | |
| 4. Has your dog been vaccinated against leptospirosis? | yes | no |
| 5a. Has your dog been abroad?  5b. If yes, in which countries and when? | yes | no |
|  |  | |
| 6a. Is your dog healthy?  6b. If no, in what way? | yes | no |
|  |  | |
| 7a. Is your dog receiving any medications?  7b. If yes, which medications? | yes | no |
|  |  | |
| 8a. Has your dog received antibiotics during the past six months?  8b. If yes, which clinic has prescribed the antibiotics | yes | no |
|  |  | |
| 9 a. Do you have other animals?  9b. If yes, what kind of animals? | yes | no |
|  |  | |
| 10. Can your dog come into contact with wild animals? | yes | no |
| 11. Are there rats in your residential area? | yes | no |
| 12. Do you live in an urban or rural area? |  | |
| 13. Does your dog spend time in the forest? | yes | no |
| 14. Does your dog drink water outside?  14b. If yes, where? (puddles, ditches, lakes, sea etc.) | yes | no |
|  |  | |
| 15. Does your dog swim outside?  15b. If yes, where? (puddles, ditches, lakes, sea etc.) |  |  |
|  |  | |
| 16. Is your dog used for hunting? | yes | no |
| 17a. Does your dog travel regularly within Sweden?  17b. If yes, where? | yes | no |
|  |  | |

Appendix 2 shows the questionnaire that was answered by the dog owners (translated from Swedish)
